# Expression of Fibroblast Growth Factor-21 in Muscle Is Associated with Lipodystrophy, Insulin Resistance and Lipid Disturbances in Patients with HIV

**DOI:** 10.1371/journal.pone.0055632

**Published:** 2013-03-22

**Authors:** Birgitte Lindegaard, Thine Hvid, Thomas Grøndahl, Christian Frosig, Jan Gerstoft, Pernille Hojman, Bente Klarlund Pedersen

**Affiliations:** 1 Centre of Inflammation and Metabolism, Department of Infectious Diseases, Rigshospitalet, Copenhagen, Denmark; 2 Copenhagen Muscle Research Centre, Rigshospitalet, Copenhagen, Denmark; 3 Department of Infectious Diseases, Rigshospitalet, Copenhagen, Denmark; 4 Institute of Exercise and Sport Sciences, Department of Human Physiology, Copenhagen, Denmark; Wageningen University, The Netherlands

## Abstract

**Background:**

Fibroblast growth factor (FGF)-21 is a novel regulator of glucose and lipid metabolism. Recently, increased FGF-21 mRNA expression in muscle was found in patients with type 2 diabetes, but the role for FGF-21 in muscle is not well understood. Patients with HIV-infection and lipodystrophy are characterised by various degree of lipid-driven insulin resistance. We hypothesized that muscle FGF-21 mRNA would be altered in HIV patients with lipodystrophy.

**Design:**

Twenty-five HIV-infected men with lipodystrophy (LD) and 15 age-matched healthy controls, received an oral glucose tolerance test and a euglycemic-hyperinsulinemic clamp (50 mU/m2/min) combined with 6,6-H_2_ glucose infusion. Muscle biopsies were obtained and FGF-21 mRNA and glycogen synthase (GS) activity were measured.

**Results:**

Subjects with HIV were insulin resistant compared with non-HIV subjects. Compared to controls, HIV subjects demonstrated a twofold increase of plasma FGF-21 from 70.4±56.8 pg/ml vs 109.1±71.8 pg/ml, respectively (p = 0.04) and an eight-fold increase in muscular FGF-21 mRNA expression (p = 0.001). Muscle FGF-21 mRNA correlated inversely with the rate of disappearance of glucose during insulin clamp (r = −0.54, p = 0.0009), and the GS fractional velocity in muscle (r = −0.39, p = 0.03), and directly with fasting insulin (r = 0.50, p = 0.0022), HOMA-IR (r = 0.47, p = 0.004), triglycerides (r = 0.60. P = 0.0001), waist-to-hip ratio (r = 0.51, p = 0.0001) and limb fat mass (−0.46, p = 0.004), but not to plasma FGF-21.

**Conclusion:**

FGF-21 mRNA is increased in skeletal muscle in HIV patients and correlates to whole-body (primarily reflecting muscle) insulin resistance, but not to plasma FGF-21. Those findings add to the evidence that FGF-21 is a myokine and may suggest that muscle FGF-21 is working in a local manner.

## Introduction

Fibroblast growth factor (FGF)-21 is a circulating hormone-like cytokine and has received much attention as a regulator for glucose and lipid metabolism in liver under fasting and ketotic conditions[1].

The majority of studies investigating the role of FGF-21 are based on animal studies. In rodents FGF-21 is produced mainly by the liver and adipose tissue. In murine models, FGF-21 is reducing plasma glucose, insulin, triglycerides, LDL and HDL cholesterol [2], [3]. Furthermore, in diet-induced obesity in mice, FGF-21 is enhancing insulin-mediated suppression of endogenous glucose production and enhancing insulin-mediated glucose uptake in skeletal muscle [3]. In lipid metabolism, FGF-21 induces ketogenesis during starvation [4], promotes fatty acid oxidation [5] and lipolysis in adipose tissue [6] in rodents.

Paradoxically, circulating FGF-21 is increased in obese nondiabetic subjects [7] and in newly diagnosed type 2 diabetes, where it correlates positively with adiposity and fasting insulin [8]. One human study has shown that plasma FGF-21 concentrations correlate with both hepatic as well as with whole-body (muscle) insulin resistance [9].

Recently, FGF-21 was identified as a novel myokine in humans [10]. An elevated muscular expression of FGF-21 was found in patients with type 2 diabetes [11] and insulin was identified as a stimulator of muscle-FGF-21 in two separate studies [10], [12]. However, little information is available on FGF-21 in muscle and its role in metabolism

A syndrome of lipodystrophy, characterised by subcutaneous fat loss, and a relative increase in central fat accumulation, develops in a subgroup of HIV patients, when treated with highly active combination antiretroviral therapy (HAART) that includes thymidine nucleoside analogues (NA) [13]. Lipodystrophy is associated with severe metabolic side effects, including dyslipidemia, hepatic insulin resistance, and lipid-driven impaired insulin-stimulated glucose uptake in muscle [14]–[16]. Recently, circulating FGF-21 was observed in HIV-infected patients, and especially those with lipodystrophy (11). This increase was, furthermore, closely associated with insulin resistance.

Since HIV patients with lipodystrophy share the same metabolic disturbances as subjects with type 2 diabetes we aimed to determine whether muscle FGF-21 mRNA is altered in these patients. The second aim of the study was to investigate the possible association of muscle FGF21 mRNA with other measures of adiposity and insulin resistance, including peripheral insulin resistance by using an insulin clamp combined with stable isotopes.

## Patients and Methods

### Patients

Twenty-three HIV-infected men were recruited from the outpatient clinic of the Department of Infectious Disease, Rigshospitalet in Copenhagen. LD was defined clinically by physical examination of peripheral lipoatrophy (defined by the presence of peripheral lipoatrophy with at least one moderate sign of fat loss in face, arms, buttocks, or legs based on a physical examination by a single investigator (BL) using a validated questionnaire developed by Carr et al. [17]. Some of the subjects have been included in a former study [18]. All patients were on a stable and effective nucleoside analogue based HAART with no changes in antiretroviral therapy during the preceding 8 weeks. Fifteen age-matched HIV-negative healthy men served as controls. Demographic data were collected for each patient: age, duration of HIV infection, duration and types of all antiretroviral therapy, weight, height, CD4 count, HIV-RNA copies. Inclusion criteria were: no signs of ongoing infections; fasting glucose under 7 mmol/L and 120 min glucose after an OGTT below 11.1 mmol/L, no dyslipidemia (triglycerides >1.7 mmol/L and/or HDL-cholesterol <0.9 mmol/L); suppressed viral load (<20 copies/mL). Exclusion criteria were: Severe cardiovascular diseases; arthritis; severe neuropathy; opportunistic infections that required hospitalisation within the last 6 weeks; diabetes (fasting glucose ≥7 mmol/L or 2-hrs. glucose >11 mmol/L after an OGTT); hepatitis C; concurrent therapy with antidiabetic agents, anticoagulant or any hormones. Fifteen age- and VO_2max_ -matched HIV-seronegative healthy men served as controls. The control subjects have been included in a former a study [18].

Written informed consent was obtained from all subjects according to the requirements from the local ethical committee and the Helsinki Declaration II, and the approval from the local ethical committee (The Ethics Committee of Copenhagen and Frederiksberg) was obtained.

### Biochemical measurements

Peripheral blood samples were obtained after an overnight fasting at 8 A.M.. Measurements of total cholesterol (mmol/L), HDL-cholesterol (mmol/L), LDL-cholesterol (mmol/L), triglycerids (mmol/L), plasma glucose (mmol/L), insulin (pmol/L), were determined immediately using routine methods.

CD4 cell counts were calculated by flowcytometry and HIV-RNA copies were measured by the Amplicor HIV Monitor (Roche Molecular Systems, Branchburg, NJ) (lower limit of dectection: 20 copies/ml).

FGF-21 was measured in plasma. Ethylenediaminetetraacetate (EDTA) was used as an anticoagulant. Plasma samples were stored at −80 C until analysed. FGF-21 was determined by enzyme-linked immunosorbent assay (ELISA) kits (Biovendor, Germany). The detection limits were 15 pg/ml and the interassay coefficient of variation was 3.9%. All samples and standards were run as duplicates and the mean of duplicates was used in the statistical analyses.

### RNA analysis and quantitative PCR analysis

RNA was extracted from 20–30 mg of skeletal muscle biopsies using TriZol (Invitrogen, Carlsbad, CA) and reverse transcribed using random hexamer primers (TaqMan reverse transcription reagents; Applied Biosystems). Real-time PCR was performed on an ABI PRISM 7900 Sequence Detection System (PE Biosystems) using TaqMan reagents (Applied Biosystems). Sequence-specific FGF-21 primer/probe sets were used for FGF-21 amplification, while predeveloped household TaqMan primer/probe sets (Applied Biosystems) were used for 18S detection. The relative expression of FGF-21 was normalized to the endogenous control and expressed as FGF-21 expression per 18S expression. The levels of 18S in the skeletal muscle were not different between groups (data not shown).

### Body composition analysis

Fat and fat-free tissue masses for the whole body, trunk and extremities were measured using dual-energy X-ray absorptiometry (DXA) scanner (Lunar Prodigy, GE Medical Systems Wisconsin, USA, version 8.8) [17]. Whole-body and regional fat measurements (trunk and extremity) were determined as previously described [18].

### Insulin sensitivity

Insulin resistance was assessed from several measurements: fasting plasma insulin, homeostasis model (HOMA-IR) [19] and area under the curve (AUC) for the insulin concentration during an 75-g oral glucose tolerance test (OGTT)

#### Euglycemic-hyperinsulinemic clamp combined with stable isotope infusion

Diet was registered two days before the clamp and participants were advised to ingest the same diet at the end-of–study visit. Subjects were admitted at 0800 h to the laboratory after an overnight fast (including HAART). An euglycemic-hyperinsulinemic clamp combined with glucose stable isotope technique was undertaken as described previously [20]. In brief, after obtaining baseline blood samples to determine background glucose enrichment a primed 16 µmol/kg constant infusion (0.22 µmol/kg/min) of [6,6-^2^H_2_]-glucose (Cambridge Isotopes Laboratories, Inc., MA, USA) was maintained for 5 hrs to determine glucose kinetics. The clamp was initiated 2.5 hrs after the start of the isotope infusion (basal condition) and continued for 2.5 hrs, adapted after [21]. Insulin (Actrapid, Novo Nordisk Insulin), 100 IU/ml was infused at a rate of 50 mU/m^2^/min (initiated with a two-step priming dose of 200 mU/m^2^/min for 5 min followed by 100 mU/m^2^/min for 5 min). Blood glucose was maintained at 5.5 mmol/L by infusion of 20% glucose enriched to 2.5% with [6,6-^2^H_2_]-glucose [22]. The infusion of [6,6-^2^H_2_]-glucose was decreased by 75% of basal infusion rate during insulin-stimulated condition to steadily maintain the plasma glucose enrichment by accounting for the expected decline in hepatic glucose production. In the morning, the hand was wrapped in a heating blanket to obtain arterialised blood samples every 10 min during the last 30 min of the basal and insulin-stimulated conditions to determine plasma glucose concentrations and tracer-to-tracee ratio.

### Glycogen synthesis activity

GS activity was measured in muscle homogenates by using a Unifilter 350 microtiter plate assay (Whatman; Frisenette, Ebeltoft, Denmark) as described by Thomas et al.[23]. Glycogen synthase activity is expressed as fractional velocity (%FV) corresponding to GS activity at a subsaturating (0.17 mM) G-6-P concentration divided by GS activity at a saturating (8 mM) concentration of G-6-P.

### Calculations

A physiological and isotopic steady state was achieved during the last 30 minutes of the basal and the insulin-stimulated conditions, so the rates of endogenous glucose appearance (R_a_) and disappearance of glucose (R_d_) were calculated as the tracer infusion rate divided by the tracer to-tracee ratio as previously described [20]. Glucose R_a_ and R_d_ are expressed per kg body weight (µmol⋅kg bwt^−1^⋅min^−^).

### Statistical analysis

Statistical calculations were performed using SAS 9.1 (USA). Data are presented as means +/− SD. *P*<0.05 was considered significant in all analyses. Rd and Ra, Insulin, triglycerides, FFA, HDL-cholesterol, LDL-cholesterol, and FGF-21 were natural log-transformed to achieve an approximate normal distribution and equal variance. Parameters between HIV-infected patients and healthy controls were compared with unpaired t-test. Pearson's correlations were used to examine the relationship between muscle FGF-21 or plasma FGF-21 levels and markers of insulin sensitivity, as well as with anthropometric parameters.

## Results

### Baseline characteristics

Demographic, body composition and biochemical data appear from Table 1. The healthy men and the HIV-infected patients were matched on age and physical activity. All subjects were categorised as sedentary-normal active. HIV-infected patients displayed a redistribution of body fat as the percentage of fat on the limb were lower and the percentage of fat in the trunk was higher compared to control subjects. The patients also had disturbances in their lipid metabolism as fasting triglycerides and total-cholesterol levels were higher, and HDL-cholesterol level was lower. In addition, compared to controls, the HIV-infected patients were characterized by peripheral insulin resistance as whole-body insulin-stimulated glucose uptake (R_d_) and incremental glucose uptake (R_d_ basal – R_d_ clamp) were decreased. Furthermore, the HIV subjects had higher basal endogenous glucose production, lower insulin-mediated suppression of endogenous glucose production (R_a_), but no difference in the incremental suppression of endogenous glucose production during the clamp (R_a_ basal – R_a_ clamp) as compared to control subjects.

**Table 1:** Baseline characteristics of patients and healthy controls.

	HIV patients (n = 23)	Healthy controls (n = 15)
Age (years)	47.9 (9.5)	47.5 (6.1)
Duration of HIV infection (years)	15.6 (9.6)	
Duration of antiretroviral therapy (years)	10.3 (4.3)	
CD4+ cell (cells/µl)	558 (208)	
LogHIV-RNA (copies/ml)	1.33 (0.12)	
**Antiretroviral use**		
NNRTI-based HAART, PI-based HAART, NNRTI-,PI-based HAART regime, No.	12/14/2	
Current Thymidine-NRTI use, No. (%)	11 (47.8)	
Current PI use, No. (%)	13 (56.7)	
Current NNRTI use, No. (%)	11 (47.8)	
**Physical activity parameters**		
VO_2max_ (LO_2_/min)	2.3 (0.5)	2.5 (0.6)
**Body composition**		
Body-mass index (kg/m^2^)	23.7 (2.9)	23.7 (1.9)
Weight (kg)	73.6 (11.2)	76.9 (7.4)
Waist (cm)	93.6 (6.4)	90 (5.7)
Waist-to.hip ratio	1.01 (0.04)	0.94 (0.03)
Fat mass (kg)	13.8 (5.3)	15.7 (4.4)
Trunk fat mass (kg)	9.8 (3.9)	8.9 (3.0)
Trunk fat percentage (%)	71.2 (6.2) ****	56.1(5.2)
Limb fat mass (kg)	3.5 (1.6)****	6.2 (1.5)
Limb fat percentage (%)	25.1 (6.1) ****	40.2 (4.9)
Trunk-to-limb fat ratio	3.09 (1.17)*	1.4 (0.29)
Lean mass (kg)	57.0 (6.8)	58.2 (5.2)
**Metabolic parameters**		
Total-cholesterol (mmol/L)	5.5 (0.9)**	4.63 (0.64)
HDL-C (mmol/L)	1.23 (0.52)*	1.51 (0.32)
LDL-C (mmol/L)	3.7 (0.9)	3.3 (0.6)
Triglycerides (mmol/L)	2.55(1.43)****	0.76 (0.24)
Glucose (mmol/L)	5.4 (0.6)	5.2 (0.3)
Insulin (pmol/L)	52 (25)****	25 (8.9)
HOMA-IR	2.2 (1.4)****	0.99 (0.37)
**Insulin sensitivity**		
Rate of appearance (µmol glucose/kg/min)		
Basal	14.2 (0.49)***	11.8 (2.0)
Clamp	6.4 (1.8)**	4.0 (2.5)
Delta†	7.8 (2.1)	7.8 (1.9)
Rate of disappearance (µmol glucose/kg/min)		
Basal	14.2 (0.49)***	11.8 (2.0)
Clamp	40.2 (9.9)**	48.6 (8.4)
Delta†	26.02 (10.1)**	36.81 (7.14)
**Glucose tolerance**		
Glucose area under the curve (mmol/Lmin)	826 (200)*	670 (126)
Insulin area under the curve (pmol/Lmin)	52360 (31017)**	23505 (10598)

Data are presented as mean (SD).

† Delta, differences between clamp and basal values. HAART, highly active antiretroviral therapy; PI, protease inhibitor; NRTI, nucleoside reverse transcriptase inhibitor; NNRTI, non- nucleoside reverse transcriptase inhibitor. HOMA-IR, homeostatic model assessment for insulin resistance, Rate of appearance and disappearance, Rate of appearance and disappearance of glucose during a euglycemic-hyperinsulinemic clamp performed in both HIV patients and healthy controls.

* *P*<0.05;

** *P*<0.01;

*** *P*<0.001,

**** *P*<0.0001 by *t*-test.

### Plasma FGF-21 and FGF-21mRNA in muscle

**Figure 1:**
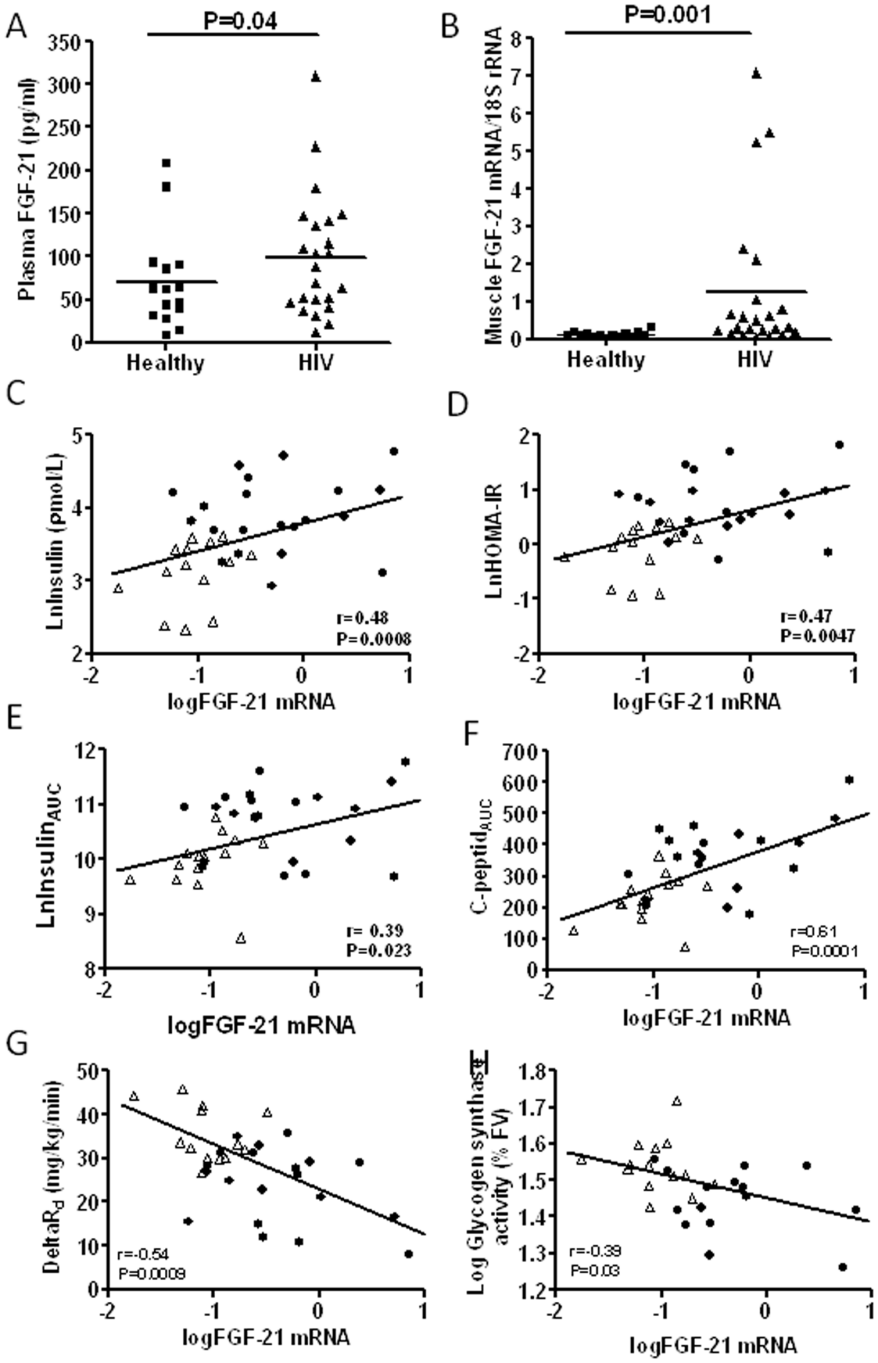
FGF-21 mRNA are increased in muscle from subjects with HIV-lipodystrophy and correlates to several measurement of insulin resistance. (A) Fasting plasma levels of fibroblast growth factor (FGF) 21 are increased 2-fold in HIV subjects with lipodystrophy compared to healthy men; (B) mRNA expression of FGF-21 are increased 8-fold in muscle biopsies from HIV subjects with lipodystrophy compared to healthy men; (C–F) Plots of FGF-21 mRNA in muscle versus several measurements of insulin resistance: FGF-21 mRNA in muscle are positively correlated to fasting insulin (C), HOMA-IR (D), Area under the curve for insulin during an oral glucose tolerance test (E), Area under the curve for C-peptide during an oral glucose tolerance test (F), and negatively correlated to the incremental rate of disappearance of glucose (G), and fractionel velocity of glycogen synthesis (H) in healthy (◊) and HIV subjects with lipodystrophy (•). In the dot plots data for each subjects are given and the line represent means. * P<0.05 and ***P<0.001 for healthy vs HIV-lipodystrophy patients. For plots, linear regression lines, correlations coefficient, and significance levels are given for all subjects.

Plasma FGF-21 was elevated in the HIV-group compared to healthy controls (70.4±56.8 pg/ml vs 109.1±71.8 pg/ml, respectively) (P = 0.04) (Figure 1A).

FGF-21 mRNA expression in skeletal muscle was increased 8-fold in patients with HIV relative to healthy individuals (P<0.0001, parametric statistics, and p = 0.0002 for non-parametric statistics) (Figure 1B). The association between plasma FGF-21 and muscle FGF-21 did not reach statistical significance (r = 0.32; p = 0.056).

### Relationships between FGF-21 mRNA, plasma FGF-21 and insulin resistance

Muscle FGF-21 mRNA correlated positively to all markers of insulin resistance: fasting insulin (r = 0.57, p = 0.0008), homeostasis model assessment (HOMA) score (r = 0.55, p = 0.001), and insulin_AUC_ (r = 0.38, p = 0.02) (Fig. 1 C–F).

To test whether the association between insulin and FGF-21 mRNA in muscle reflect an association with peripheral or hepatic insulin resistance, we performed a euglycemic-hyperinsulinemic clamp with stable isotopes. We found that muscle FGF-21 mRNA was negatively associated with the insulin-mediated glucose-uptake (Figure 1G), but not with hepatic insulin resistance (data not shown).

We did not find any association between plasma FGF-21 and parameters of insulin resistance (fasting insulin, plasma glucose, HOMA-IR, glucose _AUC_, Insulin _AUC_, R_a_ or R_d_). However, when investigating by group, plasma FGF-21 correlated positive with insulin stimulated glucose-uptake in healthy subjects but not in the HIV patients (r = 0.51, p = 0.049) (data not shown).

Previous studies have demonstrated that insulin resistance in patients with HIV-LD are associated with decreased insulin-stimulated glycogen synthase (GS) activity [24]. Therefore, we measured GS activity. The GS fractional velocity of % total GS activity was lower in HIV patients compared with healthy controls (28±1.3; 35±1.6, respectively) in the basal stage. GS fractional velocity is known to correlate positively with the glucose R_d_ [25]. As high FGF-21 mRNA in muscle is associated with low rate of disappearance of glucose, this could be linked to low GS fractional velocity in muscle. In accordance with this hypothesis, we found that high levels of FGF-21 mRNA in muscle were associated with decreased GS fractional velocity in muscle (Fig. 1H).

### Relationships between muscle FGF-21 mRNA, and fat distribution and lipids

We found a strong negative association between muscle FGF-21 mRNA and the amount of subcutaneous fat (limb fat mass) (r = −0.46; p = 0.0038) and positive association with trunk-limb-fat-ratio (r = 0.51; p = 0.001) and triglycerides (r = 0.56; p = 0.0003). FGF-21 mRNA was not associated with total fat mass or total trunk fat (data not shown).

## Discussion

The novelty and the major findings of our study is that we demonstrate for the first time that FGF-21 mRNA expression is increased in skeletal muscle in patients with HIV-LD compared to healthy age-matched men and that muscle FGF-21 mRNA correlates negatively with the rate of insulin-stimulated glucose disappearance (primarily reflecting muscle). Furthermore, increased FGF-21 mRNA expression in muscle is associated with decreased limb fat mass, increased waist-to-hip ratio and increased triglycerides.

Only three studies are published on expression of FGF-21 in muscle in humans [10], [12], [26] and very little is known about the function of muscle FGF-21. Our result is in agreement with a previous study, in which FGF-21 mRNA was found to be increased in muscle from subjects with type 2 diabetes and the expression was increased by hyperinsulinemia [10]. However, this study did not distinguish between hepatic and peripheral insulin sensitivity as we do in the present study. Vienberg *et al*. [12] also find that FGF-21 mRNA was expressed in muscle, but they find no activation of muscle FGF-21 mRNA by a short term high fat overfeeding. In the study by Mashili *et al*. [26], FGF-21 mRNA was expressed in skeletal muscle, but no difference was found between obese subjects with normal glucose tolerance and obese subjects with Type 2 diabetes. However, in this study no healthy non-obese subjects were included.

A body of evidence show that in murine models FGF-21 lowers blood glucose, insulin, triglycerides, fat mass and increases insulin sensitivity [2], [3]. In humans, circulating FGF-21 is increased in obesity and in subjects with type 2 diabetes [7], [8]. Therefore, increased levels of circulating FGF-21 have been suggested to be a compensatory mechanism due to insulin resistance or a sign of FGF-21 resistance. In murine models, chronic administration of FGF-21 increases insulin-mediated glucose uptake in muscle, but only in diet induced obesity [3]. Furthermore, triglycerides deposit in muscle by FGF-21 administration is attenuated. Therefore, it has been suggested that the effect of FGF-21 on muscle is secondary to other events e.g. lipid toxicity in muscle.

Besides the strong correlation between insulin-mediated glucose uptake and FGF-21 mRNA in muscle, FGF-21 mRNA was also tightly correlated to triglycerides. Patients with HIV lipodystrophy have increased lipolysis and high triglycerides as wells as an increased amount of intra myocellular lipid accumulation [27]. We have previously shown that HIV patients display lipid driven insulin resistance [20]. Therefore, increased FGF-21 in muscle may be a compensatory mechanism to offset the lipid-induced insulin resistance in muscle. This explanation is supported by *in vitro* models, where FGF-21 is found to protect human myotubes from palmitate-induced insulin resistance [28], and to increase basal and insulin-stimulated glucose uptake in human myotubes [26].

In rodents, FGF21 has emerged as a hormone involved in energy homeostasis in the liver. We found lower fractional glycogen synthase activity in muscle in the patients indicating reduced capacity for deposition of glucose into glycogen in the muscle during basal condition. Furthermore, FGF-21 mRNA was associated with low fractional glycogen synthase activity in muscle. Therefore, in accordance to the role of FGF-21 in the liver in rodent, muscular FGF-21 in humans may be a marker of the substrate and energy levels in the muscle. Since our observations are cross-sectional in nature it is not possible to establish cause-and-effect relationship.

In line with FGF-21 as an energy sensor, the increased FGF-21 mRNA in muscle may be speculated to be related to mitochondrial dysfunction. Mice with late-onset mitochondrial myopathy show induction of FGF-21 in their muscles, which is closely related to the number of COX-negative muscle fibres [29], suggesting that mitochondria deficiency in skeletal muscle induces FGF21 expression. In skeletal muscle, HIV patients treated with NRTI have a reduced mitochondrial/nucleus DNA ratio, more frequent mtDNA deletions and possibly more COX-deficient muscle fibres than HIV-negative controls [30]. In addition, mitochondrial aging was recently found to be accelerated by anti-retroviral therapy through the clonal expansion of mtDNA mutations [31]. Therefore, the increase in FGF-21 could also be related to mitochondrial changes in the muscle.

Very little information on FGF receptors in humans exists. FGF21 acts through the interaction with specific FGF receptors and a cofactor called β-Klotho [32]. FGFR1 in adipose tissue has been suggested as the main receptor/tissue to serve to mediate the effect of FGF21 *in vivo* in rodents [33]. A very recent paper has shown a dramatically reduction of β-klotho mRNA in adipose tissue in HIV-patients with and without lipodystrophy [33], supporting the hypothesis that the increase in muscle FGF-21 mRNA may in part be a compensatory mechanism to the reduced FGF-21 signalling in adipose tissue and that adipose tissue may be target tissue for FGF21 in HIV-patients. A FGF-21 receptor in muscle has not yet been defined. As mentioned, FGF21 has direct effects in enhancing skeletal muscle glucose uptake in both rodents and human myocytes [3], [26] and protects human myotubes from palmitate-induced insulin resistance [28], demonstrating that FGF-21 signals directly in muscle.

In agreement with Domingo *et al.* [34], we found increased circulating FGF-21 in HIV patients with lipodystrophy. However, in contrast to their study we did not find a correlation between plasma FGF-21 and measures of insulin resistance (fasting insulin, HOMA-IR, area under the curve for insulin during an OGTT and rate of glucose disappearance during an euglycemic-hyperinsulinemic clamp combined with stable isotopes). The explanation for this discrepancy is difficult to understand. The subjects in the study by Domingo *et al.* included naive, HAART- treated patients with or withour lipodystophy and a control group. The HIV study groups were comparable with regards to the range of circulating FGF-21, age, CD4 count and BMI. The HIV-patients in our study were more insulin resistant (HOMA 2.2 vs 1,7 in the study by Domingo *et al.* ), had a higher trunk-to-limb ratio (3.09 vs 2.42), and had more hyperlipidemia (TC 5.5 vs 5.07, Triglyceriedes 2.55 vs 5.00) than the HIV subjects with lipodystrophy in the study by Domingo *et al.* In our study no patients were co-infected with hepatitis C or had chronic hepatitis B virus infection, whereas this was the case for 7.6% and 8.9% , respectively in the patients in the study by Domingo *et al*. In the study by Domingo *et al*, circulating FGF-21 was tightly correlated with markers of liver function such as AST, ALT and GGT. We did not measure AST, ALT or GGT and can therefore not speculate whether this could explain the discrepancy between the studies.

We did not find a significant correlation between high levels of circulating FGF-21 levels and FGF-21 mRNA in muscle, indicating that muscle is not the major source of circulating FGF-21. As previously suggested some evidence exists that liver may be the primary source of circulating FGF-21. The lack of association between circulating and muscle-expressed FGF-21 also suggests that muscle FGF-21 primarily works in a local manner regulating glucose metabolism in the muscle and/or signals to the adipose tissue in close contact to the muscle.

Our study has some limitations. The number of subjects is small and some correlations could have been significant with greater statistical power. Another aspect is that protein levels of FGF-21 were not determined in the muscles extracts, consequently we cannot be sure the increase in FGF-21 mRNA is followed by increased protein expression.

In conclusion, we show that FGF-21 mRNA is increased in skeletal muscle in HIV patients and that FGF-21 mRNA in muscle correlates to whole-body (primarily reflecting muscle) insulin resistance. These findings add to the evidence that FGF-21 is a myokine and that muscle FGF-21 might primarily work in an autocrine manner.
