# Correction: Expression of Fibroblast Growth Factor-21 in Muscle Is Associated with Lipodystrophy, Insulin Resistance and Lipid Disturbances in Patients with HIV

**DOI:** 10.1371/journal.pone.0127249

**Published:** 2015-04-23

**Authors:** Birgitte Lindegaard, Thine Hvid, Thomas Grøndahl, Christian Frosig, Jan Gerstoft, Pernille Hojman, Bente Klarlund Pedersen

The work described in this article does not come under the remit of the MYOAGE project by the Commission of the European Communities, and as a result, the MYOAGE grant was incorrectly included under the Funding statement for the article. The Funding statement should be revised to read as below:

“The Centre of Inflammation and Metabolism (CIM) is supported by a grant from the Danish National Research Foundation (# 02-512-55). This study was further supported by the Danish Medical Research Council (# 22-04-0588), the Lundbeck Foundation, the Danish AIDS Foundation, the Novo Nordisk Foundation, Direktør Emil Hertz og Hustru Inger Hertz' Fond, Direktør Jacob Madsen og Hustru Olga Madsens Fond, Fonden for Lægevidenskabens fremme. CIM is part of the UNIK Project: Food, Fitness and Pharma for Health and Disease, supported by the Danish Ministry of Science, Technology and Innovation. CIM is a member of DD2—the Danish Center for Strategic Research in Type 2 Diabetes (the Danish Council for Strategic Research, grant no. 09–067009 and 09–075724). The Copenhagen Muscle Research Centre is supported by a grant from the Capital Region of Denmark. The funders had no role in study design, data collection and analysis, decision to publish, or preparation of the manuscript.”
